# ERK/PP1a/PLB/SERCA2a and JNK Pathways Are Involved in Luteolin-Mediated Protection of Rat Hearts and Cardiomyocytes following Ischemia/Reperfusion

**DOI:** 10.1371/journal.pone.0082957

**Published:** 2013-12-30

**Authors:** Xin Wu, Tongda Xu, Dongye Li, Shasha Zhu, Qiuping Chen, Wenjing Hu, Defeng Pan, Hong Zhu, Hong Sun

**Affiliations:** 1 Institute of Cardiovascular Disease Research, Xuzhou Medical College, Xuzhou, Jiangsu, PR China; 2 Department of The First Clinical College, Nanjing Traditional Chinese Medicine University, Nanjing, Jiangsu, PR China; 3 Department of Physiology, Xuzhou Medical College, Xuzhou, Jiangsu, PR China; Loyola University Chicago, United States of America

## Abstract

Luteolin has long been used in traditional Chinese medicine for treatment of various diseases. Recent studies have suggested that administration of luteolin yields cardioprotective effects during ischemia/reperfusion (I/R) in rats. However, the precise mechanisms of this action remain unclear. The aim of this study is to confirm that luteolin-mediated extracellular signal regulated kinase (ERK1/2) and c-Jun N-terminal kinase (JNK) pathways are responsible for their cardioprotective effects during I/R. Wistar rats were divided into the following groups: (i) DMSO group (DMSO); (ii) I/R group (I/R); (iii) luteolin+I/R group (Lut+I/R); (iv) ERK1/2 inhibitor PD98059+I/R group (PD+I/R); (v) PD98059+luteolin+I/R group (PD+Lut+I/R); and (vi) JNK inhibitor SP600125+I/R group (SP+I/R). The following properties were measured: contractile function of isolated heart and cardiomyocytes; infarct size; the release of lactate dehydrogenase (LDH); the percentage of apoptotic cells; the expression levels of Bcl-2 and Bax; and phosphorylation status of ERK1/2, JNK, type 1 protein phosphatase (PP1a), phospholamban (PLB) and sarcoplasmic reticulum Ca^2+^-ATPase (SERCA2a). Our data showed that pretreatment with luteolin or SP600125 significantly improved the contraction of the isolated heart and cardiomyocytes, reduced infarct size and LDH activity, decreased the rate of apoptosis and increased the Bcl-2/Bax ratio. However, pretreatment with PD98059 alone before I/R had no effect on the above indexes. Further, these consequences of luteolin pretreatment were abrogated by co-administration of PD98059. We also found that pretreatment with PD98059 caused a significant increase in JNK expression, and SP600125 could cause ERK1/2 activation during I/R. In addition, we are the first to demonstrate that luteolin affects PP1a expression, which results in the up-regulation of the PLB, thereby relieving its inhibition of SERCA2a. These results showed that luteolin improves cardiomyocyte contractile function after I/R injury by an ERK1/2-PP1a-PLB-SERCA2a-mediated mechanism independent of JNK signaling pathway.

## Introduction

Myocardial ischemia/reperfusion (I/R) is a complex pathophysiological event, which can result in serious acute or chronic myocardial damage [1], eventually leading to myocardial ultrastructural changes and remodeling as well as myocardial systolic and diastolic dysfunction. Therefore, preventing the diminished function of cardiomyocytes may play a more important role in the recovery from ischemia/reperfusion injury (IRI). However, the intracellular signaling pathways participating in the process of I/R have not been completely clarified, previous studies have shown that PI3K/Akt pathway promoted survival in adult rat cardiomyocytes during I/R, not only exhibited the action of anti-apoptosis, but also involved in the regulation of myocardial contractility [2]–[3]. Many studies have also suggested that the mitogen activated protein kinases (MAPKs) might be important regulator of apoptosis in response to myocardial I/R. The MAPK family consists of three well-characterized subfamilies: extracellular signal regulated kinases (ERKs), c-Jun N-terminal kinases (JNKs) and p38 [4]. ERK1/2 and JNK have been studied extensively in recent years, but reports on their precise roles on cardiomyocytes during I/R were still conflicting [5]–[9].

Luteolin (3′, 4′, 5′, 7′-tetrahydroxyflavone) is a common flavonoid that exists in many types of plants, including fruits, vegetables and medicinal herbs. Preclinical studies have shown that luteolin possesses a variety of biological and pharmacological activities [10]–[11]. In recent years, studies have suggested that luteolin inhib-it lipoteichoic acid -induced ERK 1/2, p38, and JNK phosphorylation in H9c2 cells, the phenomenon has also been found in human gingival fibroblasts [12], [13]. Furthermore, our previous research result showed that administration of luteolin can improve the function of adult rat myocardium following I/R via signaling through the PI3K/Akt pathway [2]. However, the myocardial protective effects of luteolin against IRI and the mechanisms by which it affects the ERK1/2 and JNK family have not been clarified. We therefore attempted to elucidate whether the ERK/JNK signaling pathways are involved in mediating the protective effects of luteolin during I/R.

At present, numerous reports have shown that type 1 protein phosphatase (PP1a) is an important negative regulator of cardiac function [14], [15]. PP1a is present in the sarcoplasmic reticulum (SR) and it is the main phosphatase that dephosphorylates phospholamban (PLB), thereby impacting the activity and activity of cardiac sarcoplasmic reticulum Ca^2+^-ATPase (SERCA2a) [16]. Early studies found that PLB regulates the activity of the Ca^2+^-ATPase pump on the SR. Clearly, the activation of SERCA2a depends on the phosphorylation of PLB at Ser16 and Thr17, which can relieve its inhibitory effect on SERCA2a and allow for increased Ca^2+^ pumping into the SR [17]. As such, PLB plays a major role in modulating cardiac function, and it is further indicated that PP1a is the main phosphatase responsible for dephosphorylating PLB. As a consequence, PP1a may also be a critical regulator of cardiac contractility. Furthermore, PP1 is under the regulation of two endogenous proteins, inhibitor-1 and inhibitor-2 [18], [19]; however, it is not currently clear whether there are any other additional endogenous proteins that modify PP1a. Our previous studies suggest that pretreatment of adult rat cardiomyocytes with luteolin significantly increases the expression of p-PLB and SERCA2a via the PI3K/Akt pathway [2]. In this study, we focus on whether ERK/JNK mediates the effects of luteolin and if the improvement in the contractile function of cardiomyocytes is related to PP1a. This investigation, for the first time, uncovered a new anti-IRI property of luteolin.

## Materials and Methods

### Animals and reagents

These experiments received prior approval by the Animal Ethics Committee of the Xuzhou Medical College (permit number CMCACUC2009-04-135). Adult male Wistar rats (clean grade, Xuzhou Medical College, China) weighing 220–250 g were randomly allocated into the following groups: (i) DMSO group (DMSO); (ii) I/R group; (iii) luteolin+I/R group (Lut+I/R); (iv) ERK1/2 inhibitor PD98059+I/R group (PD+I/R); (v) PD98059+luteolin+IR group (PD+Lut+I/R); and (vi) JNK inhibitor SP600125+I/R group (SP+I/R). Luteolin (purity >98%) was purchased from Sigma-Aldrich (Fluka; Germany), dissolved in dimethyl sulfoxide and then diluted with buffer or culture medium to a final concentration of 0.01%, which itself had no effect on the heart [20]. PD98059 and SP600125 were purchased from Cell Signaling Technology Inc. (USA).

### Heart perfusion and experimental preparation

Ten minutes after being heparinized with sodium heparin (1000 U/kg) by intraperitoneal injection, male Wistar rats were anesthetized with sodium pentobarbital (150 mg/kg). The heart was rapidly excised and placed into ice-cold Krebs-Henseleit (KH) buffer solution containing the following (mmol/l): 120 NaCl, 4.7 KCl, 1.2 KH_2_PO_4_, 1.2 MgSO_4_, 25 NaHCO_3_, 11 glucose and 1.25 CaCl_2_. The aorta was cannulated, immediately mounted onto a Langendorff heart perfusion apparatus, and retrogradely perfused with KH buffer, with the pressure kept constant at 80 mmHg. The KH solution was bubbled with 95% O_2_ and 5% CO_2_ in an atmosphere at 37°C. After all hearts were equilibrated, the different groups were subjected to the following treatments. In the DMSO group (n = 6), hearts wereperfused with KH buffer and DMSO for 210 min. In the I/R group (n = 6), hearts were perfused with KH buffer for 60 min, then subjected to global ischemia for 30 min and reperfused for 120 min with KH solution. In the luteolin pretreatment group (n = 6), hearts were perfused with KH buffer for 30 min prior to pretreatment with 40 µmol/l luteolin for 30 min, and then underwent the I/R procedure described above. In the PD98059 pretreatment group (n = 6), PD (20 µmol/l) was administered for 30 min after hearts were perfused with KH buffer for 30 min, followed by the same I/R procedure described above. In the PD+Lut+I/R group (n = 6), hearts were perfused with PD at 20 µmol/l for 30 min before administration of luteolin, and then subjected to the same course as the Lut+I/R group. In the SP+I/R group (n = 6), SP (10 µmol/l) was administered prior to ischemia (30 min) and reperfusion (120 min) as stated above. Luteolin and inhibitors were infused into the heart via a side pipe located just proximal to the heart cannula. KH buffer was perfused at the beginning of normoxic perfusion. The concentrations of the drugs used were selected following preliminary experiments.

### Measurement of cardiac function

Left ventricular systolic and diastolic function was continuously monitored before and during the entire I/R procedure with a Biopac system (Biopac), with a Millar transducer instrument (pressure sensor) inserted into the left ventricle through the left atrium as described previously [21]. During the procedure, heart rate (HR), left ventricular systolic pressure (LVSP), left ventricular end-diastolic pressure (LVEDP) and the rate of the rise and fall of ventricular pressure (+dp/dt, −dp/dt) were recorded. Then, a hemodynamic analysis system (Chengdu, China) was used to calculate left ventricular function parameters.

### Determination of infarct size

Infarct size was measured by triphenyltetrazolium chloride (TTC) (Sigma-Aldrich; USA) staining as described previously [22]. Briefly, at the end of reperfusion, the left ventricle was frozen and kept at −20°C. Two hours later, the ventricular tissue was sliced into 2-mm sections across the long axis (beginning from cardiac apex) and placed in 1% TTC for 20 min at 37°C; specimens were then incubated in a 10% formaldehyde solution for 1 h. The infarct region was stained gray, whereas the normal zone stained brick red. Isolation of myocardial tissue for weight measurement: the percentage of infarcted area in the total left ventricle was measured by weighing the infarcted tissue and the whole left ventricle tissue.

### Measurement of LDH activity in coronary effluent

After heart reperfusion for 10 min, about 10 ml of coronary effluent was collected from each group for determination of LDH activity, which was measured by spectrophotometry and calculated according to the manufacturer's instructions (Jiancheng Bioengineering Institute; Nanjing, China). Measurements were repeated three times for each group.

### Evaluation of apoptotic cell number

Apoptosis was detected with a terminal deoxynucleotidyl transferase-mediated dUTP nick end labeling (TUNEL) kit (Roche; Switzerland). Following to the manufacturer's instructions, at least three myocardial tissue sections were chosen from each group. Cells were examined by light microscopy (200× magnification), with 10 fields was observed and each viewed field containing at least 50 cells. Moreover, 4′,6-diamidino-2-phenylindole (DAPI) was used for staining all nuclei of cardiomyocytes. With the TUNEL method, only the nuclei of apoptotic cells stained brown, while normal nuclei stain blue with DAPI. The ratio of TUNEL-positive cardiomyocytes was calculated as follows: number of apoptotic cells/total number counted ×100%.

### Isolation of cardiomyocytes

Ventricular myocytes were isolated from the Wistar rats as we have described previously [2], [21]. In brief, cardiomyocytes were isolated from the heart using the collagenase (type II) purchased from Invitrogen (USA) and Ca^2+^-free buffer. The cells were then suspended three times in Krebs-bicarbonate solution (pH 7.2) containing the following (mmol/l): 15 NaCl, 85 KCl, 30 KH_2_PO4, 5 MgSO4, 5 sodium pyruvate, 5 creatine, 20 taurine, 2 L -glutamic acid, 20 HEPES, 20 glucose, 0.2 CaCl_2_ and 0.5 EGTA, gassed with 100% O_2_ at 37°C. After isolation, 80%–87% of the viable myocytes were quiescent; myocytes were subsequently cultured in Dulbecco's minimal essential medium (DMEM) containing 1% penicillin-streptomycin at a density of 2×10^4^ cells in 12-well culture dishes. Finally, the myocytes were placed in a carbon dioxide incubator (Heraeus; Germany) with an atmosphere of 95% O_2_ and 5% CO_2_ at 37°C.

### Simulated I/R protocol

After cardiomyocytes were equilibrated for 1 h in the incubator, cardiac myocytes were exposed to an ‘ischemic buffer’ that contained the following (mmol/l): 118 NaCl, 24 NaHCO_3_, 1.0 NaH_2_PO_4_, 2.5 CaCl_2_-2H_2_O, 1.2 MgCl_2_, 20 sodium lactate, 16 KCl and 102 deoxyglucose (pH adjusted to 6.2) as reported previously [23]. The cells were incubated at 37°C in a tri-gas incubator with a 1% O_2_ and 5% CO_2_ atmosphere for 3 h during the entire simulated ischemic period. The ischemia buffer was then placed in a normal cell medium under normoxic conditions during the 2-h reperfusion process.

### Treatment of cells with luteolin, PD98059 and SP600125

Initially, cardiomyocytes were pretreated with a series of different concentrations of luteolin (2, 4, 8, 16 µmol/L). The optimal concentration of luteolin (used in the remainder of the experiments) was determined based on a trypan blue exclusion assay, LDH release in the medium and cardiac myocyte shortening amplitude. In the luteolin and SP600125 (10 µmol/L) groups, cells were cultured with luteolin at a concentration of 8 µmol/L for 12 h, or with SP600125 for 30 min before the simulated I/R (sI/R) procedure. In the luteolin+PD98059 group, PD98059 (10 µmol/L) was added to the cells 30 min before the luteolin pretreatment, which was then followed by sI/R. For DMSO group, cardiomyocytes were incubated with vehicle alone for the remainder of the experiment.

### Measurement of the shortening amplitude of myocytes

After each group (except for the DMSO group) completed the reperfusion phase, a few drops of medium containing ventricular myocytes were added to an open chamber on the stage of an inverted microscope (Olympus; Japan). After the cells spontaneously attached to the bottom of the chamber, cardiomyocytes were superfused at 2 ml/min with KH buffer (containing 2.0 mM Ca^2+^ and 100 nM isoprenaline) at 37°C, adjusted to pH 7.4 by equilibration, with a 95% O_2_ and 5% CO_2_ atmosphere. Isoprenaline increased the shortening amplitude of myocytes in a concentration-dependent manner, and 0.1 M isoprenaline exerted the maximal effect [20]. Some rod-shaped ventricular myocytes with clear sarcomeres were chosen to undergo electrical stimulation at 0.5 Hz. At least 10 cardiomyocytes per heart from each group were evaluated. The whole procedure was recorded by a video recorder (Panasonic; Japan) as previously described [20], [24], and the output of the video edge detector was sent to a computer. Ventricular myocardial contraction was indexed by the percent reduction in resting cell length following stimulation.

### Western blot analysis

After 2 h reperfusion, cardiomyocytes were washed with PBS. The cells were harvested and homogenized in lysis buffer supplemented with proteinase inhibitor, and placed on ice for 30 min. After centrifugation at 20,000 g for 20 min at 4°C, the supernatant was collected and protein concentrations were measured using a modified Bradford assay (Bio-Rad, CA, USA). Whole lysates (40 µg) were resolved by 8–12% sodium dodecyl sulfate–polyacrylamide gel electrophoresis (SDS-PAGE); proteins were then transferred to polyvinylidene difluo-ride (PVDF) membranes. After blocking with 5% non-fat dry milk in Tris-buffered saline containing 0.1% Tween 20 (TBST), membranes were immunoblotted overnight at 4°C with primary antibodies against the following: Bcl-2 and Bax (1∶500, Santa Cruz, USA); ERK1/2, phospho-ERK1/2, JNK, phospho-JNK, PP1a, phospho-PP1a, PLB, and phospho-PLB (1∶1000; Cell Signaling Technology, MA, USA); SERCA2a (1∶5000; Abcam; England); and β-actin (1∶1000; Zhongshan; Beijing, China). This step was followed by incubation with the corresponding secondary antibodies (1∶1000; Zhongshan; Beijing, China) at room temperature for 1 h. Protein bands were visualized by nitro blue tetrazolium and 5-bromo-4-chloro-3-indolyl-phosphate. The membranes were scanned, the images transferred to a computer, and the relative intensity of bands was analyzed by the Image J 3.0 system (National Institutes of Health; MD, USA). The optical density of the bands of the control group was considered to be 1 arbitrary densitometry unit.

### Statistical analysis

One-way and two-way ANOVA was conducted across all groups, followed by a Bonferroni post-hoc correction between all group comparisons. Statistical analysis was performed with Graph Pad Prism 5 software, and the data were expressed as means ± SEM. A difference was considered significant at a level of *P*<0.05.

## Results

### Determination of best luteolin pretreatment concentration

**Figure 1:**
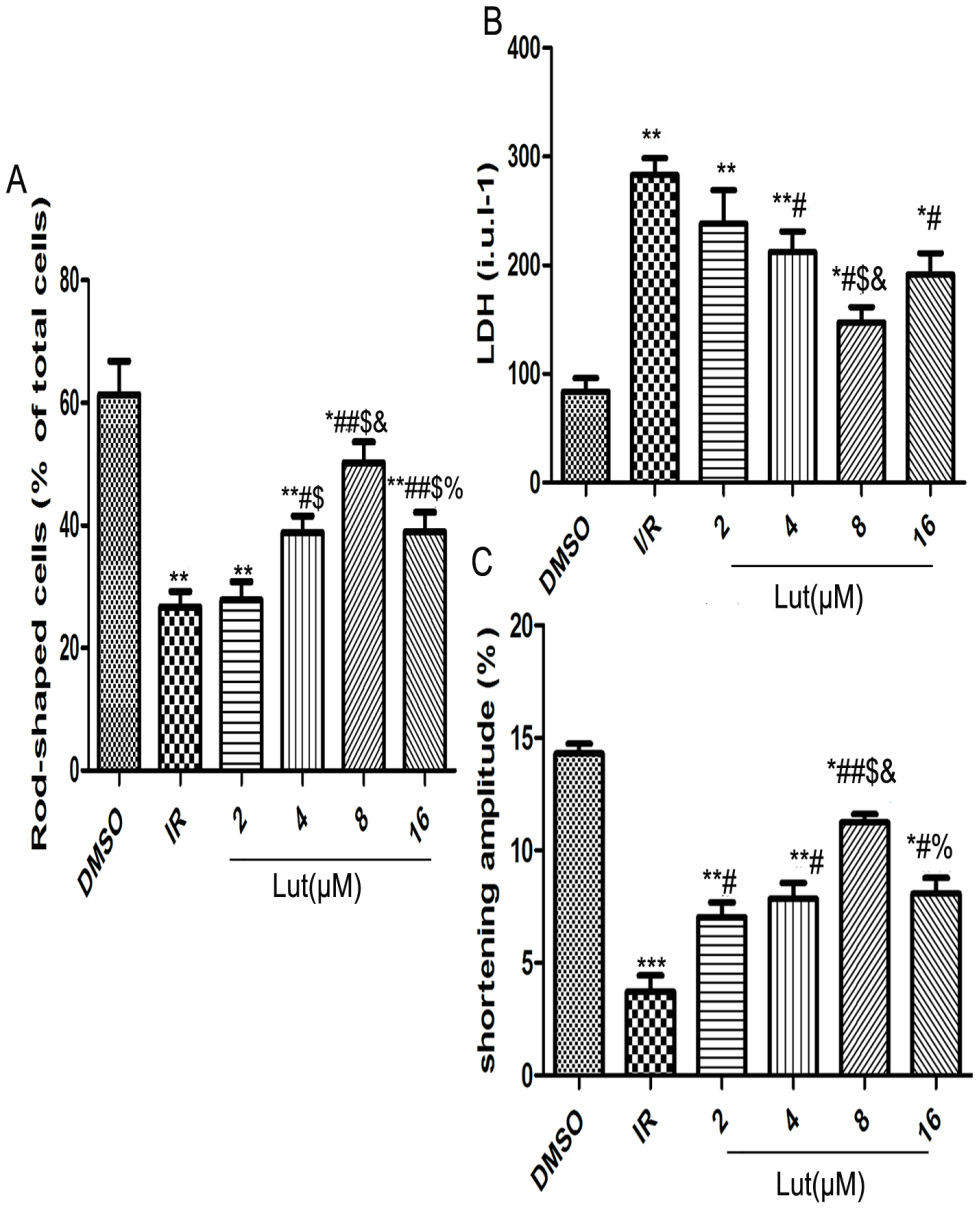
Grope the best concentrations of luteolin. Different concentration of luteolin on the number of rod-shaped cells(%) (A), Release of LDH (B) and cardiomyocyte shortening amplitude (C) after I/R were detected. Each value represents the mean ± SEM, n = 6. *P<0.05,**P<0.01,***P<0.001 *versus* DMSO, ^#^ *P*<0.05, ^##^ *P*<0.01 *versus* I/R. ^$^ *P*<0.05 versus Lut(2 µM)+I/R, ^&^ *P*<0.05 versusu Lut(4 µM)+I/R, ^%^ *P*<0.05 versusu Lut(8 µM)+I/R.

Trypan blue staining revealed that necrotic cells stained blue and were round-shaped, while normal cardiac myocytes were rod-shaped and unstained. The percentage of rod-shaped cells was decreased in the I/R group compared with the DMSO group (26.70±1.45% vs 61.37±3.15%, *P*<0.01). After treatment with 2, 4, 8 and 16 µmol/l of luteolin before I/R, the percentage of cells surviving was increased (27.83±1.72%, 38.83±1.59, 50.27±1.95, 38.93±1.87%). The rate of rod-shaped cells was not statistically different between the 2 µmol/l luteolin group and the I/R group (*P*>0.05). The percentage of rod-shaped cells was increased in the 4 and 16 µmol/l luteolin groups (all *P*<0.05). However, the rate of rod-shaped cells was significantly increased in 8 µmol/l luteolin group (*P*<0.01), and was statistically different among all dosage groups (*P*<0.05) (Fig. 1A).

I/R significantly increased the release of LDH into the cardiomyocyte cultured medium compared with the DMSO group (238.10±8.81 vs 83.76±7.09 *P*<0.01). Pretreatment with 2 µmol/l of luteolin had no obvious effect compared to the I/R group (238.10±17.87 vs 238.10±8.81, *P*>0.05). However, the effect of pretreatment was improved by increasing the concentration of luteolin to 4, 8 and 16 µmol/L (238.10±8.81 vs 212.00±10.98, 147.6±7.98, 191.6±17.27, *P*<0.05); when luteolin was administered at a concentration of 8 µmol/l, the I/R-induced increase in LDH release was markedly limited (*P*<0.05), and this effect was statistically different compared to the 4 µmol/L group (*P*<0.05) (Fig. 1B).

As is shown in Fig. 1C, the shortening amplitude of myocytes in the I/R group was markedly diminished compared to the DMSO group (3.73±0.72% vs 13.50±0.83%, *P*<0.001). Luteolin inhibited the decrease of cardiomyocyte shortening amplitude after I/R (6.14±0.53, 8.14±0.77, 11.12±0.41, and 9.94±0.50% in luteolin 2, 4, 8 and 16 µmol/l groups, respectively) compared with the I/R group (*P*<0.05 in 2, 4, 16 µmol/l groups). When luteolin was administered at a concentration of 8 µmol/l, the I/R-induced decrease in shortening amplitude was maximally inhibited (*P*<0.01) (Fig. 1C).

The above results demonstrated that luteolin, at the concentration of 8 µmol/l, significantly inhibits cardiomyocyte necrosis and enhances myocyte shortening amplitude compared to other groups; thus, we employed this dose in our following experiments.

### Effects of luteolin and SP600125 on the function of isolated hearts subjected to I/R

Before ischemia, there were no significant differences between groups in each of the experiment parameters. However, as Table 1 shows, the I/R group exhibited a decrease of HR, LVSP and ±dp/dt, and an increase in LVEDP, compared to the DMSO group. Pretreatment with either luteolin or SP600125 before I/R significantly improved cardiac systolic/diastolic function and increased HR. Application of PD98059 alone did not exert this effect. In addition, administration of PD98059 before luteolin markedly reversed the beneficial effects of luteolin on these parameters of myocardial function.

**Table 1:** Each group of Myocardial Function in Isolated Ischemia/Reperfused hearts.

		DMSO	I/R	PD+I/R	Lut+I/R	PD+Lut+I/R	SP+I/R
HR(Beats/min)	**a**	267.20±12.35	259.40±10.82	265.00±10.18	265.60±11.57	263.60±13.96	270.40±11.69
	**b**	260.60±11.44	140.40±9.27***	143.20±8.29***	222.80±11.24* #	181.00±9.96** # $	220.60±0.64* # &
	**c**	255.60±11.08	133.40±9.42***	136.20±7.70***	213.80±11.28* #	174.00±1.66** # $	214.20±0.50* # &
LVSP(mmHg)	**a**	128.60± 7.70	129.00± 7.87	127.00±7.81	124.80±7.51	127.60±7.94	126.80±6.69
	**b**	124.60±7.37	46.80±4.84***	46.60±5.65***	94.00±5.36* #	69.60±5.35** # $	97.00±6.47* # &
	**c**	119.60±7.41	41.40±4.32***	41.60±5.85***	91.20±5.25* #	65.20±6.00** # $	90.80±6.98* # &
LVEDP(mmHg)	**a**	11.02±0.49	11.90±0.65	11.32±0.47	11.46±0.81	11.12±0.73	11.28±0.57
	**b**	11.30± 0.31	20.72±1.24***	19.94±0.92***	14.14±0.60* #	17.34±0.30** # $	14.38±0.60* # &
	**c**	11.92± 0.51	21.04±1.15***	20.54±0.95***	14.88±0.57* #	18.12±0.47** # $	15.18±0.49* # &
+dp/dt(mmHg/s)	**a**	2764.20± 153.38	2763.80±158.42	2761.80±161.63	2746.60±157.00	2727.00±160.00	2782.00±151.50
	**b**	2774.20±174.10	1242.40±107.17***	1267.00±117.97***	2242.60±139.00* #	1728.80±105.62** # $	2289.60±117.27* # &&
	**c**	2706.40±142.18	1068.00±92.34***	1064.40±81.61***	2031.80±145.56* #	1560.40±82.12** # $	2172.60±117.94* # &&
−dp/dt(mmHg/s)	**a**	2683.20±176.25	2623.60±156.45	2602.40±168.88	2614.40±176.47	2691.20±154.13	2634.00±128.86
	**b**	2583.00±15.50	1159.00±132.87***	977.80± 106.87***	2198.80±146.89* #	1669.00±108.94** # $	2198.40±109.69* # &
	**c**	2495.40±144.18	1067.40±128.77***	906.00±102.12 ***	1973.80±141.09* #	1484.40±114.90** # $	2097.00±105.78* # &

Effects of luteolin on various parameters of hemodynamic measurements during a reperfusion period in isolated working rat heart preparations. The results are expressed as the mean ± SEM, n = 6.

*P*<0.05,

*P*<0.01,

P<0.001 versus DMSO;

^#^ *P*<0.05 versus I/R;

$ *P*<0.05 versus I/R+Lut (40 µM);

& P<0.05,

&& P<0.01 versus I/R+Lut (40 umol/L)+PD (20 umol/L).

a indicate baseline, b and c indicate perfusion 30 min and 120 min in the DMSO group, but in other groups, b and c indicate reperfusion 30 min and 120 min after ischemia respectively.

### Effects of Luteolin and SP600125 on myocardial infarct size and LDH activity in coronary effluent

**Figure 2:**
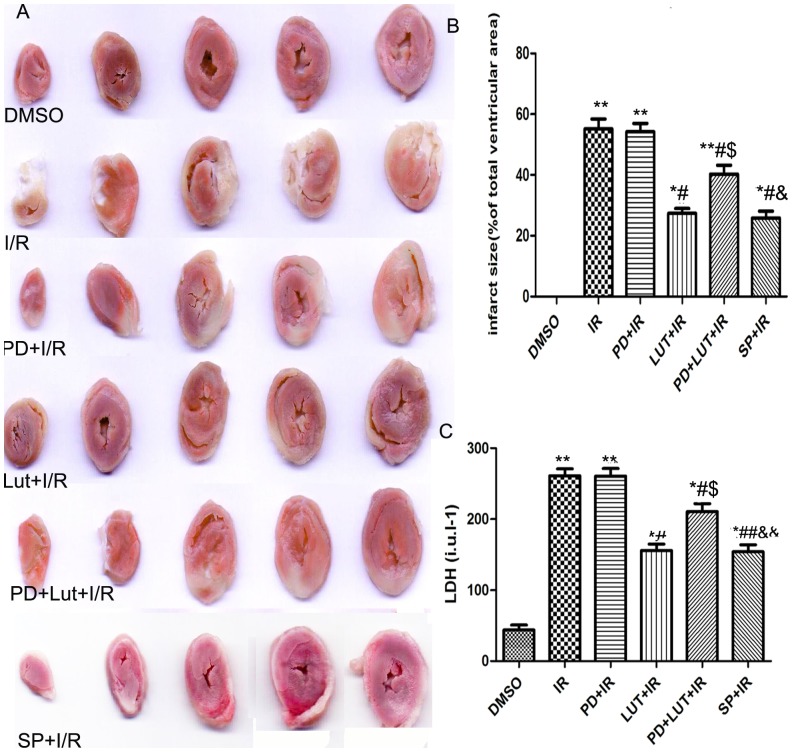
Infarct size and LDH release quantity of each group. Effects of luteolin and SP600125 on infarct size (A, B) and release of LDH (C) in isolated I/R hearts. The results are expressed as the mean ± SEM, n = 6. *P<0.05, **P<0.01 versus DMSO; #P<0.05 versus I/R; $P<0.05, versus I/R+Lut (40 µM), &&P<0.01, &P<0.05 versus I/R+Lut(40 µmol/L)+PD (20 µmol/L).

Representative images of myocardial tissue from each group are shown in Figure 2. Infarct size was expressed as the percentage of infarct area/total area. I/R significantly increased infarct size (55.17±3.29% vs 0.00±0.00%, P<0.001) compared to the DMSO group. Luteolin or SP600125 administration significantly decreased infarct size (27.34±1.66, 25.83±2.65 vs 55.17±3.287%, *P*<0.05) compared to the I/R group. Treatment with PD98059 did not yield any significant effect on infarct size compared with the I/R group (54.21±2.76% vs 55.17±3.287%, *P*>0.05). However, pretreatment with PD98059 before luteolin administration can reverse the effect of luteolin (40.30±2.93% in PD+Lut+I/R group vs 27.34±1.66 in Lut+I/R group, *P*<0.05).

Luteolin and SP600125 can also attenuate the release of LDH, which is an indicator of myocardial injury after I/R. LDH, a marker of necrosis, significantly decreased after administration of luteolin or SP600125, compared with the I/R group (155.80±9.18, 154.10±9.88 vs 261.10±9.68, *P*<0.01). The effect of luteolin was abolished by co-administration of PD600125 (210.52±11.30 in PD+Lut+I/R group vs 155.80±9.18 in Lut+I/R group, *P*<0.05). No significant difference in the release of LDH was found between the PD+I/R and I/R groups.

### Luteolin and SP600125 inhibit I/R-induced apoptosis

**Figure 3:**
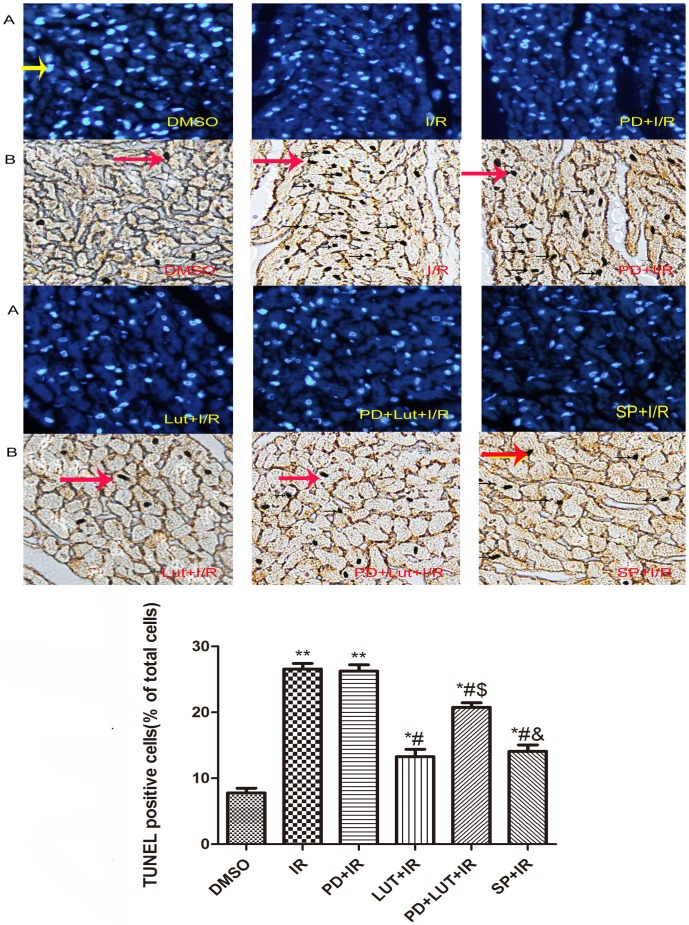
Apoptosis of each group. A representative photomicrograph of a TUNEL (B) and DAPI-stained (A) cardiomyocytes were showed. After 2-apoptotic effect of luteolin and SP600125 by TUNEL staining. The results are expressed as the mean ± SEM, n = 3. ^#^ *P*<0.05 versus I/R; ^$^ *P*<0.01 versus I/R+Lut (40 µM), ^&^ *P*<0.05 versus I/R+Lut(40 µmol/L)+PD (20 µmol/L). Cells were examined by light microscopy (200× magnification). Yellow allow indicate DAPI-stained nucleus, red allows indicate TUNEL-positive caryons.

I/R-induced myocyte apoptosis was demonstrated by TUNEL/DAPI staining (Figure 3). Compared with the DMSO group, the percentage of TUNEL-positive cardiomyocytes was increased (7.78±0.72% vs 26.59±0.82, P<0.01). The percentage of TUNEL-positive cardiomyocytes was significantly lower in the luteolin and SP600125 groups compared to the I/R group (13.28±1.12%, 14.08±0.97% vs 26.59±0.82%, P<0.05). However, the effect of luteolin was largely abrogated by PD98059 (20.77±0.68% in PD+Lut+I/R group vs 13.28±1.12% in Lut+I/R group, P<0.05). Administration of PD98059 alone had no effect on the level of apoptosis induced by I/R (*P*>0.05).

### Effects of luteolin and SP600125 on the shortening amplitude of isolated ventricular myocytes

The above results indicated that endogenous ERK1/2 was not activated by I/R. However, luteolin preconditioning significantly activated the ERK1/2 pathway, and this played an important role in the cardioprotective effects of this molecule. Therefore, we did not include the PD+IR group in following experiment.

**Figure 4:**
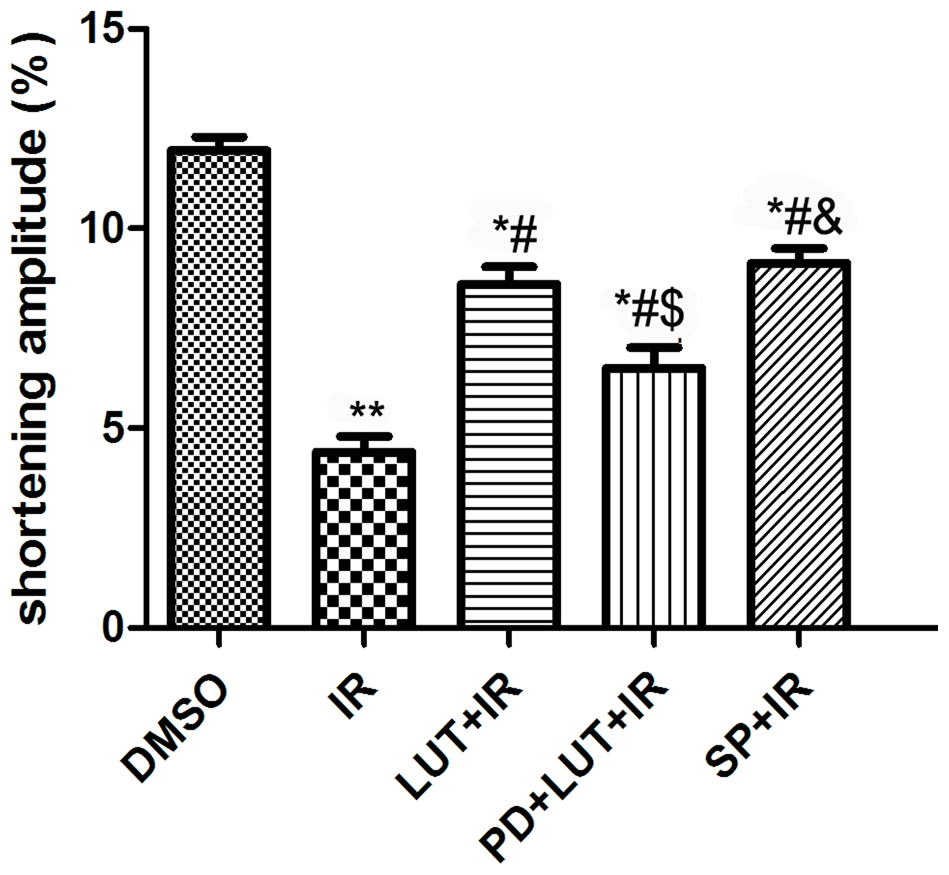
The contractile function of single cardiac cell. Effect of luteolin and SP600125 on shortening. amplitude in isolated I/R cardiomyocytes were observered. The results are expressed as the mean ± SEM, n = 3. *P<0.05, **P<0.01 versus DMSO; ^#^ *P*<0.05 versus I/R; ^$^ *P*<0.05 versus I/R+Lut (8.0 µM), ^&^ *P*<0.05 versus I/R+Lut(8.0 µM)+PD(10 µM).

We next examined the protective effects of luteolin on single cardiomyocytes. Our results showed that I/R markedly decreases the shortening amplitude of myocytes compared to the DMSO group (4.41±0.39 vs 11.96±0.33, *P*<0.01). Administration of either luteolin or SP600125 significantly blunted the reduction of shortening amplitude caused by IRI (8.60±0.45, 9.13±0.37 vs 4.41±0.39, *P*<0.05), while co-administration of PD98059 abrogated the effect of luteolin (6.51±0.51 vs 8.60±0.45, *P*<0.05) (Figure 4).

### Luteolin and SP60012 increase the expression of p-ERK1/2 and Bcl-2 while decreasing the expression of p-JNK and Bax

**Figure 5:**
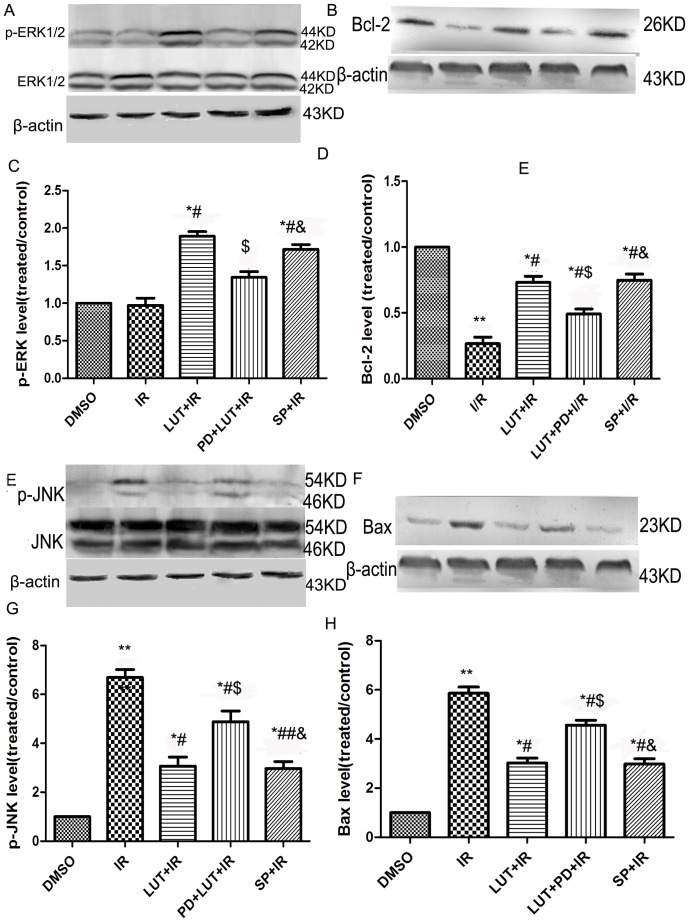
The expression of p-ERK1/2, p-JNK, Bcl-2 and Bax. The effects of luteolin and SP600125 on the expression of total ERK and p-ERK (A, B), Bcl-2 (C, D) total JNK and p-JNK (E, F), Bax (G, H). After 2 h reperfusion, the myocytes were harvested to detect protein expressions by western blot analysis. The results were expressed as the mean ± SEM. n = 3. ^**^ *P*<0.01 versus DMSO; ^#^ *P*<0.05, ^##^ *P*<0.01 versus I/R; ^$^ *P*<0.05 versus I/R+Lut (8.0 µM), ^&^ *P*<0.05, ^&&^ *P*<0.01 versus I/R+Lut(8.0 µM)+PD(10 µM).

As shown in Fig. 5, the expression of Bcl-2, Bax, ERK1/2 and JNK were examined by Western blot analysis in order to explore whether the ERK1/2 and JNK signaling pathways are involved in the anti-apoptotic effect of luteolin on cardiomyocytes. Compared with the I/R group, the phosphorylation of ERK at Thr202 (44 kDa) and Tyr204 (42 kDa) were increased, while the phosphorylation of JNK at Thr183 (46 kDa) and Tyr 185 (54 kDa) were reduced, after pretreatment with luteolin (*P*<0.05) and SP600125 (*P*<0.01). Co-pretreatment with luteolin and PD98059 resulted in the abrogation of the luteolin-induced ERK1/2 activation and JNK inactivation, as compared with the Lut+I/R group (*P*<0.05); however, there were no differences in total ERK1/2 and JNK expression among the groups studied.

The I/R-induced apoptosis of cardiomyocytes has been shown to be dependent on the activation of Bax and inactivation of Bcl-2. To study the involvement of the ERK1/2 and JNK pathways in the anti-apoptotic effect of luteolin on cardiomyocytes, we used inhibitors of JNK and ERK1/2 in the following assays. I/R markedly reduced Bcl-2 levels while inducing Bax expression, as compared to the DMSO group (*P*<0.01). Pretreatment with luteolin or an inhibitor of JNK reversed the result (*P*<0.05) as compared to the I/R group. However, when luteolin pretreatment was conducted in the presence of PD98059, the positive effects of luteolin were almost completely abolished (PD+Lut+I/R group vs Lut+I/R group, *P*<0.05) (Figure 5).

### Effects of Luteolin and SP600125 on PP1a, PLB and SERCA2a protein expression in cardiomyocytes

**Figure 6:**
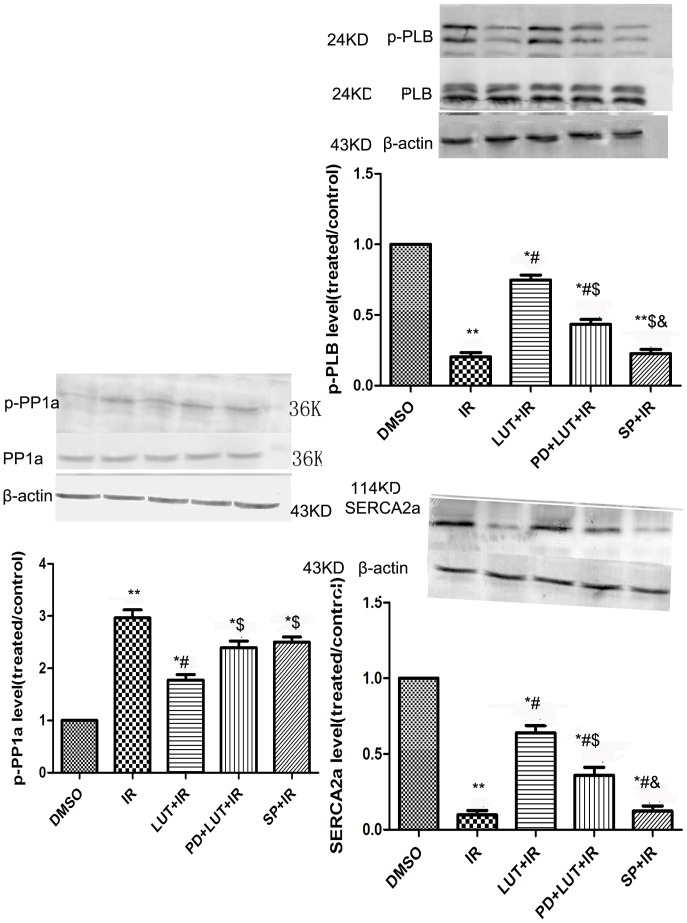
The expression of p-PP1a, p-PLB and SERCA2a. The effects of luteolin and SP600125 on the expression of total PP1a and phospho-PP1a (A, B), total PLB and phospho-PLB (C, D), SERCA (E, F). The results were expressed as the mean ± SEM, n = 3. ^*^ *P*<0.05,^**^ *P*<0.01 versus DMSO; ^#^ *P*<0.05 versus I/R; ^$^ *P*<0.05 versus I/R+Lut (8.0 µM); ^&^ *P*<0.05 versus I/R+Lut(8.0 µM)+PD(10 µM).

Western blotting analysis revealed that levels of SERCA2a and phospho-PLB were increased, while the phosphorylated forms of PP1a (PP1) were reduced, in the luteolin pretreatment group compared with the sI/R group (*P*<0.05) (Figure 6). No significant difference was detected between the SP600125 treated group and the I/R group. The effects of luteolin on the above protein were also reversed by the prior administration of PD98059 (PD+Lut+I/R group vs Lut+I/R group, *P*<0.05).

## Discussion

It is well understood that many pathways are involved in the process of IRI. To better understand the importance of pathways activated during I/R, we studied the activation of ERK1/2 and JNK during I/R-induced injury by application of inhibitors PD98059 and SP600125. Our results have demonstrated that I/R mainly activates JNK, rather than the ERK1/2 pathway. JNK is a key factor in the signal transduction leading to myocardial injury after I/R; in contrast, ERK1/2 is not activated during I/R. However, several recent studies [25]–[27] have found that ERK is activated during the ischemia and reperfusion stages, and was shown to be required for survival signaling. This difference observed between our study and the aforementioned previous reports may be due to differences in the cell types and experimental protocols employed. Though ERK1/2 and JNK belong to one family, they do not necessarily work in concert. In the aforementioned research, the authors employed a protocol involving 30 min of ischemia and 45 min of reperfusion, which is drastically different from our protocol of 3 h of ischemia and 2 h of reperfusion. On the other hand, it is possible that the activations of JNK reached its maximal levels or the activation period of ERK1/2 may have been missed. These differences in experimental design might explain the contrasting results.

In the present study, we investigated the protective effects of luteolin against IRI and explored the mechanisms involved by employing an ex vivo rat model of I/R [28], [29]. Our results showed that I/R leads to the apoptosis of cardiomyocytes, the release of LDH in coronary effluent, and decrease of contractile function and infarction of myocardial tissue. Pretreatment with luteolin or SP600125 significantly attenuated I/R-induced cardiomyocyte death, LDH leakage and infarct size. Further, it also improved the systolic/diastolic function of single cardiomyocytes and whole heart, with a concomitant increase in p-ERK1/2 and decrease in p-JNK expression. All these effects of luteolin were abolished by ERK1/2 inhibition. Moreover, luteolin effectively improved the degeneration of cell shortening amplitude and cell death induced by I/R, with a maximal effect observed at a dose of 8 µmol/l. This suggests that luteolin may actually exert anti-IRI effects in myocytes at this concentration.

Luteolin is a widely distributed flavonoid, and possess a variety of biological and pharmacological activities. Moreover, luteolin is a potential candidate for the prevention and treatment of cardiovascular diseases. In our previous study, we reported that administration of luteolin prior to I/R improves the contractility of cardiomyocytes and inhibit apoptosis through the PI3K/Akt pathway [2]. However, the mechanism by which it exerts cardioprotection against IRI has not been fully elucidated. In the present study, we examined the role of JNK and ERK1/2 in the cardioprotection provided by luteolin during I/R. We showed that pre-ischemic treatment with luteolin reduces infarct size, decreases the levels of apoptosis of cardiomyocytes and LDH leakage, and partially preserves heart/single cardiomyocyte function following I/R. Our results are in agreement with previous reports that the neuroprotective effects of luteolin involve signaling through the MAPK pathway [30], [31]. Gutiérrez et al. have also shown that luteolin can inhibit LPS- and LTA-induced ERK1/2, p38 and JNK phosphorylation in human gingival fibroblasts and embryonic ventricular myocardial H9c2 cells [12], [13].

Several investigators have reported that apoptosis is the more critical consequence of myocardial injury in I/R animal models [32], [33]. Recent studies indicate that the ERK1/2 and JNK pathways are important mediators of apoptosis induced by stressful stimuli [5], [34]. Our present results clearly demonstrate that luteolin pretreatment increases the level of anti-apoptotic protein Bcl-2 while also decreasing the abundance of pro-apoptotic protein Bax, thereby increasing the Bcl-2/Bax ratio. These effects of luteolin demonstrate that it acts at least partly via the ERK1/2 and JNK signaling pathways. In this study, we used both TUNEL assays and monitoring of apoptosis regulators Bcl-2/Bax to investigate the anti-apoptosis effect of luteolin via the ERK1/2 and JNK pathways.

We employed inhibitors of ERK1/2 and JNK to clarify the interplay and cross-talk between ERK1/2 and JNK. Our present findings clearly demonstrate that the inhibition of JNK signaling results in an increase in ERK1/2 activation. A similar result obtained in the luteolin pretreatment group, with the activation of ERK1/2 inhibiting JNK expression. These results are in agreement with previous reports [35], [36] that the inhibition of ERK1/2 in human alveolar macrophages reduces DUSP16 levels, leading to an increase in JNK phosphorylation. Further, the activation of JNK inhibited the ERK1/2 signaling pathway in mouse cardiomyocytes. This interplay between ERK1/2 and JNK pathways may serve as part of the defense mechanisms of the cardiomyocytes in response to stress.

**Figure 7:**
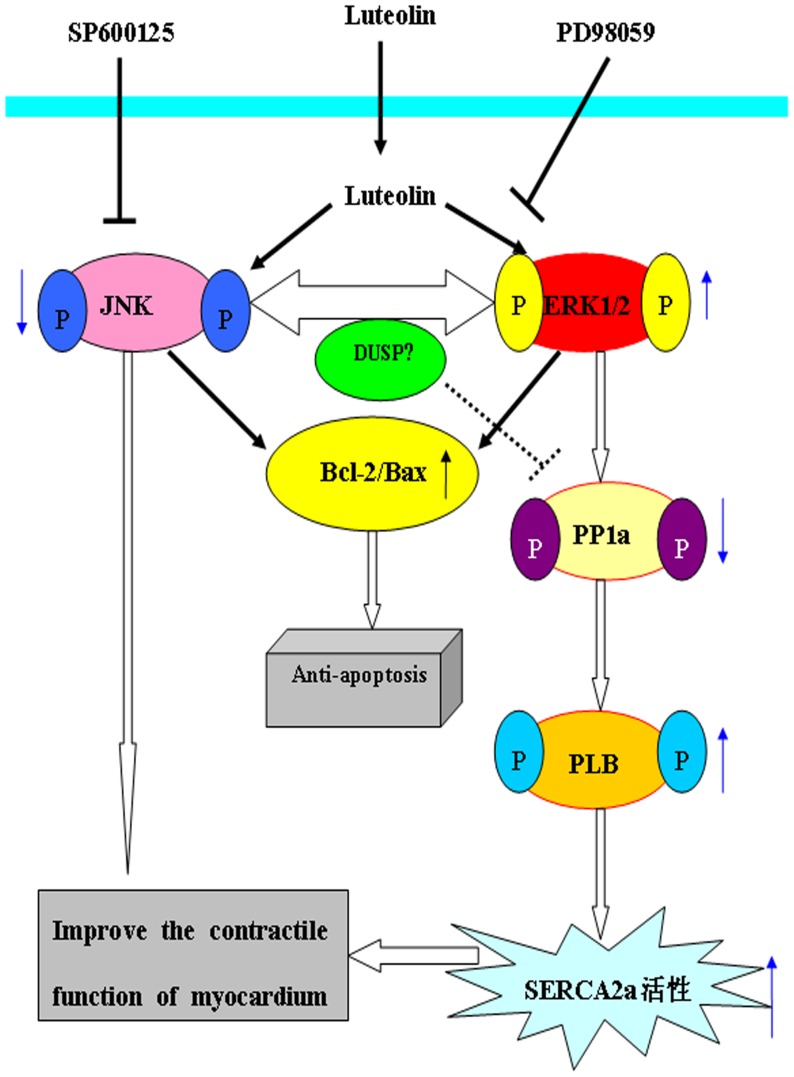
The possible mechanisms of luteolin exerting its protective effects on myocardium following I/R injury. Pretreatment with luteolin and SP600125 can deregulate the expression of p-JNK, upregulate the expression of p-ERK following I/R, which can result in cells apoptosis were inhibited and contractile function of myocardium was improved. Pretreatment with luteolin can also decrease the expression of SERCA2a via ERK1/2-PP1a-PLB pathway. The effect of luteolin was almost completely abolished by pretreatment PD98059 before it.

In addition, we have also found that administration of luteolin significantly attenuated the expression of PP1a, which is a particularly important negative regulator of cardiac function. Our previous studies have shown that luteolin markedly improves the contractile function of cardiomyocytes by increasing the expression of phospho-PLB and SERCA2a [2]; thus, we further explored the mechanisms by which luteolin regulates the activation of p-PLB and SERCA2a. SERCA2a is a central regulator of cardiac function, and overexpression of SERCA2a has been shown to enhance cardiac contraction and relaxation [37]. The activation of SERCA2a is mediated via the phosphorylation and dephosphorylation of PLB. PP1 is a particularly important negative regulator of cardiac function, as it dephosphorylates PLB and has significant impacts on SERCA2a activity and cardiac performance. It is well understood that activation of the ERK1/2 and JNK pathways transduce signals initiated by various extracellular stimuli and signaling pathways [38], [39]. Previous reports have shown that the activation of JNK plays an important role in I/R-induced apoptosis [34], while the activation of ERK1/2 is a central mediator of cell survival and preventing apoptosis [5]. In contrast, little is known about the involvement of ERK1/2 in improving the contractile function of cardiomyocytes during I/R. In this study, we examined how the ERK1/2 pathway mediates the positive effects of luteolin on the contractile function of cardiomyocytes and the whole heart. We found that luteolin markedly inhibited phospho-PP1a levels via activation ERK1/2, and thereby increased the expression of phosphor-PLB and SERCA2a (Figure 7). In contrast, JNK inhibitor SP600125 could activate ERK1/2, but did not affect the expression of PP1a, PLB and SERCA2a. This indicates that there are other proteins located in the JNK and ERK1/2 pathway that have an impact on PP1a. Our findings shed further light on how luteolin regulates the contractile function of I/R-injured cultured rat cardiomyocytes and isolated hearts via the ERK1/2 pathway. Celia et al [40] have shown that the activation of PP1 depends on MEK, a protein upstream of ERK1/2 in neuronal cells, which is similar to our results in cardiomyocytes. Our results are not consistent with the study of Monick et al [41], which demonstrated that ERK1/2 inhibited the expression of JNK and further activated PP1a. This could be explained by the different cell type we used. However, additional investigations are required to delineate the real interaction between ERK1/2 and PP1a.

In conclusion, this study provides evidence supporting the hypothesis that JNK and ERK1/2 play essential roles in regulating the effects of luteolin on myocyte contractility and apoptosis. Activation of ERK1/2 and inhibition of JNK have potential therapeutic applications in protecting against I/R-mediated cardiomyocyte dysfunction. In addition, our data suggest that luteolin increases the phosphorylation of PLB and up-regulates SERCA2a via the ERK1/2-PP1a signaling pathway. This suggests that luteolin provides effective cardioprotection against myocardial IRI.
